# The Impact of Chitinase Binding Domain Truncation on the Properties of *Ca*Chi18B from *Chitinilyticum aquatile* CSC-1

**DOI:** 10.3390/md23030093

**Published:** 2025-02-20

**Authors:** Chenxi Gu, Jianrong Chen, Xinyue Huang, Yongqiang Jiang, Na Ou, Dengfeng Yang, Mingguo Jiang, Lixia Pan

**Affiliations:** 1 Guangxi Key Laboratory for Polysaccharide Materials and Modifications, School of Marine Sciences and Biotechnology, Guangxi Minzu University, Nanning 530008, China; nonnetta427@163.com (C.G.); huangxinyue1110@163.com (X.H.); 2 National Key Laboratory of Non-Food Biomass Energy Technology, Guangxi Key Laboratory of Marine Natural Products and Combinatorial Biosynthesis Chemistry, Guangxi Academy of Marine Sciences, Guangxi Academy of Sciences, Nanning 530007, China; 17861506571@163.com (J.C.); dengfengyang@163.com (D.Y.); 3 Institute of Biology, Guangxi Academy of Sciences, Nanning 530007, China; jxc28@sina.com (Y.J.); ouna_18577071692@126.com (N.O.)

**Keywords:** N-acetyl-D-glucosamine, chitinase binding domain, bi-functional chitinase, enzymatic properties

## Abstract

The chitinase binding domain (ChBD) plays a crucial role in the properties of enzymes. To assess its impact, we cloned a truncated mutant of the chitinase gene *CaChi18B* from the novel chitinase-producing facultative anaerobic bacterium *Chitinilyticum aquatile* CSC-1, designated as *CaChi18B_ΔChBD_s_*. The recombinant chitinase was successfully expressed and purified, exhibiting a specific activity of 3.48 U/mg on colloidal chitin, with optimal conditions at 45 °C and pH 6.0, and retaining over 80% activity at temperatures up to 40 °C. Kinetic analysis revealed that the *K_m_* value was 1.159 mg mL^−1^ and the *V_max_* was 10.37 μM min^−1^ mg^−1^. Compared to *Ca*Chi18B_ΔChBD_1_, which has only the first ChBD truncated at the N-terminus, *Ca*Chi18B_ΔChBD_s_ exhibited minor changes in the optimal temperature and pH, while the *K_m_* and *V_max_* values increased significantly. *Ca*Chi18B_ΔChBD_s_ exhibited tolerance to various metal ions, with K^+^ and NH_4_^+^ enhancing activity, while Cu^2+^ significantly inhibited it. Most organic reagents had minimal impact, except for formic acid, which severely reduced activity. The primary hydrolysis product in the initial phase was GlcNAc, contrasting with (GlcNAc)_2_ for *Ca*Chi18B_ΔChBD_1_. These findings indicated that the ChBD influences the enzyme’s *K_m_*, *V_max_*, and product distribution, enhancing our understanding of ChBD’s roles and advancing chitin utilization.

## 1. Introduction

Chitin is a high-molecular-weight polysaccharide composed of N-acetyl-D-glucosamine (GlcNAc) molecules linked by β-1,4-glycosidic bonds. It is the second most abundant natural organic polymer in nature, surpassed only by cellulose [1]. As the largest ecosystem on Earth, the ocean harbors chitin as one of the most abundant organic carbon sources. The degradation and metabolism of chitin by microorganisms play crucial roles in driving marine material cycles [2]. However, the staggering amount of chitin waste derived from marine sources, reaching up to 10^12^ to 10^14^ tons annually [3,4], poses significant challenges. Improper disposal of this waste not only results in resource wastage but also poses potential environmental threats. Therefore, the efficient utilization of chitin resources has become a focal point of current research.

Chitin’s poor solubility in water, dilute acids, and dilute alkalis limits its direct application. However, the degradation products of chitin, particularly chito-oligosaccharides (NCOS) and N-acetyl-D-glucosamine (GlcNAc), exhibit excellent water solubility, biocompatibility, and bioactivity [5]. NCOS possesses a wide range of biological activities, including anti-tumor [6], antioxidant [7], antibacterial [8], immunostimulatory [9], and gut microbiota-regulating properties [10]. Meanwhile, GlcNAc demonstrates anti-inflammatory [11,12] and antiviral properties [13], and serves as a glucosamine supplement [14]. Consequently, these degradation products hold broad application prospects and significant market potential in fields such as biomedicine, food processing, agricultural production, and environmental protection [15,16].

Chitinase (EC 3.2.1.14) is a class of glycoside hydrolases capable of hydrolyzing the β-1,4-glycosidic bonds in chitin. Based on their mechanisms of action, chitinases can be classified into endochitinases and exochitinases [17,18]. Endochitinases randomly cleave the β-1,4-glycosidic bonds within the chitin molecule, producing NCOS and GlcNAc [18,19], while exochitinases are further divided into chitobiosidases and β-N-acetylglucosaminidases (GlcNAcase, EC 3.2.1.52). Chitobiosidases act on the reducing or non-reducing ends of chitin, sequentially releasing (GlcNAc)_2_, whereas β-N-acetylglucosaminidases hydrolyze (GlcNAc)_2_ into GlcNAc [20].

These enzymes are widely distributed in bacteria, fungi, plants, animals, and even humans [21]. Bacterial chitinases have garnered significant attention due to their high efficiency and ease of genetic manipulation [22]. To date, various chitinases have been isolated and characterized from bacteria such as *Bacillus cereus* [23], *Chitinibacter* sp. GC72 [24], *Laceyella putida* [25], and *Massilia timona* [26]. Furthermore, numerous chitinase genes have been successfully cloned and heterologously expressed in *Escherichia coli*, demonstrating high expression levels and an ease of purification [27,28].

In biotechnology and industrial production, chitinase holds significant potential for the degradation of marine chitin waste, such as shrimp and crab shells, as well as for the production of its derivatives, including NCOS and GlcNAc. Some exochitinases are bifunctional enzymes that exhibit both chitobiosidase and β-N-acetylglucosaminidase activity. Due to their ability to produce a more uniform product, GlcNAc, these bi-functional enzymes are often more appealing for industrial applications than single-function chitinases, as they can simplify the processes of separation and purification. For example, the bi-functional enzyme derived from *Aeromonas caviae* CHZ306 produces over 90% GlcNAc from powdered chitin within 6 h [29]. Similarly, the chitinase from *Paenibacillus* sp. TKU052 possesses multiple catalytic functions, exhibiting exochitinase, endochitinase, and β-N-acetylglucosaminidase activities. This enzyme can efficiently convert colloidal chitin into GlcNAc, achieving a yield of 94.35 to 98.60% within 12 to 24 h [30]. As a result, these bi-functional and multi-functional chitinases are increasingly prominent in industrial applications.

Based on amino acid sequence similarity, chitinases are primarily classified into glycoside hydrolase families GH18 and GH19 in the carbohydrate-active enzymes (CAZy) database [31]. Notably, bacterial chitinases predominantly belong to the GH18 family [32]. Chitinases in the GH18 family adopt the classic (β/α)_8_ TIM barrel catalytic structure, consisting of eight parallel β-strands surrounded by eight α-helices [15]. In addition to the core catalytic domain (CD), GH18 chitinases often contain auxiliary domains such as chitin-binding domains (ChBDs) and fibronectin type III (FN-III) domains [33,34,35]. Studies have shown that ChBDs not only enhance the ability of chitinases to degrade crystalline chitin but also significantly influence their thermal stability [34]. For instance, a heterologous ChBD from *Pyrococcus furiosus* has been shown to markedly improve the thermal stability of other chitinases [36]. Therefore, investigating the role of ChBDs in chitinase function not only aids in optimizing enzymatic performance but also provides theoretical support for the development of highly efficient chitinases [37].

In our previous work [38], we successfully expressed a soluble chitinase, *Ca*Chi18B_ΔChBD_1_, by truncating the ChBD_1_ domain of the full-length chitinase *Ca*Chi18B. This enzyme exhibited a specific activity of 3.05 U/mg on colloidal chitin, with optimal activity at 50 °C and pH 7.0. It also demonstrated bi-functional enzymatic activity, possessing both chitobiosidase and β-N-acetylglucosaminidase activity [38]. To further investigate the role of ChBD_2_ in chitinase function, we constructed the *Ca*Chi18B_ΔChBD_s_ variant by truncating the ChBD_2_ domain of *Ca*Chi18B_ΔChBD_1_ and heterologously expressed it in *E. coli* BL21-CodonPlus (DE3)-RIPL. Through chitin-binding assays and enzymatic property analyses, we systematically studied the effects of ChBD_2_ on enzyme activity, thermal stability, pH adaptability, kinetic parameters, and product distribution. This research aims to provide a theoretical foundation for the functional optimization and application development of chitinases.

## 2. Results

### 2.1. Bioinformatics Analysis

**Figure 1:**
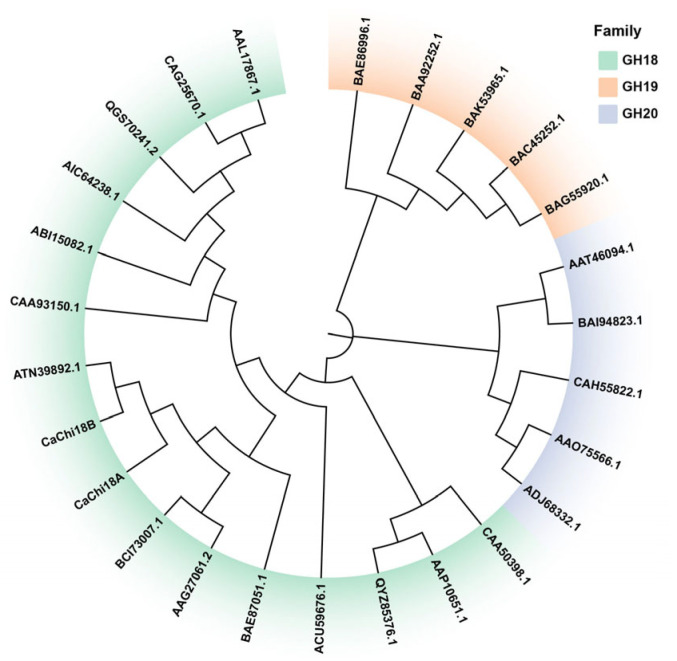
Sequence analysis of the recombinant chitinase *Ca*Chi18B.

**Figure 2:**
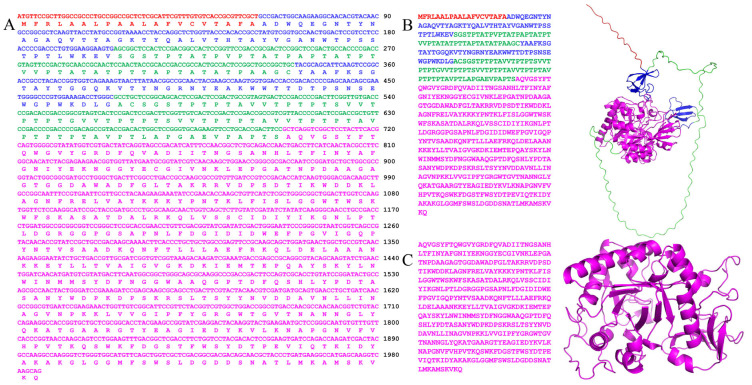
Sequences and three-dimensional structural model of *Ca*Chi18B. (**A**) Nucleotide sequence and amino acid sequence of *Ca*Chi18B. (**B**) and (**C**) show the amino acid sequences and predicted three-dimensional structural models of *Ca*Chi18B and *Ca*Chi18B_ΔChBD_s_, respectively. Red represents the signal sequence, blue represents the chitinase binding domains (ChBDs), and magenta represents the core catalytic domain (CD).Multiple sequence alignment analyses of the amino acid sequences of the two ChBD domains in *Ca*Chi18B revealed that one proline (P38) in ChBD_1_ was highly conserved. Additionally, four aromatic amino acid residues (W3, Y9, Y22, and W45) and five uncharged non-polar amino acid residues (G12, V15, G19, A24, and P38) were also highly conserved (Figure 3). In ChBD_2_, five amino acids were highly conserved (Y10, G13, V16, Y23, and P33). Additionally, four aromatic amino acid residues (Y10, Y18, Y23, and W41) and five uncharged non-polar amino acid residues (G13, V16, G20, A25, and P33) were also highly conserved.

According to previous bioinformatics analyses [38], phylogenetic tree analysis indicated that the chitinase *Ca*Chi18B belongs to the GH18 family (Figure 1). Additionally, sequence alignment revealed that *Ca*Chi18B exhibits low similarity with other reported GH18 family chitinases. The highest similarity was observed with the multi-functional chitinase ATN39892.1 from *Chitinolyticbacter meiyuanensis* SYBC-H1, at 69.83% [39], followed by the chitinase *Ca*Chi18A from *C. aquatile* CSC-1, with a similarity of 67.08% [40]. In contrast, the similarity with the chitinase ACU59676.1 from *Chitinophaga pinensis* DSM 2588 was significantly lower, at only 26.21% [41]. ChBD_1_ was located between amino acids 20 and 68 (Ala20 to Val68), ChBD_2_ was found between amino acids 114 and 157 (Tyr114 to Leu157), and the catalytic domain (CD) spanned amino acids 233 to 647 (Gln233 to Asp647) (Figure 2). Therefore, the open reading frame (ORF) of the constructed chitinase *Ca*Chi18B_ΔChBD_s_ was 1242 bp, encoding 414 amino acids, with a theoretical molecular weight of 47 kDa and a theoretical isoelectric point (pI) of 5.85.

**Figure 3:**
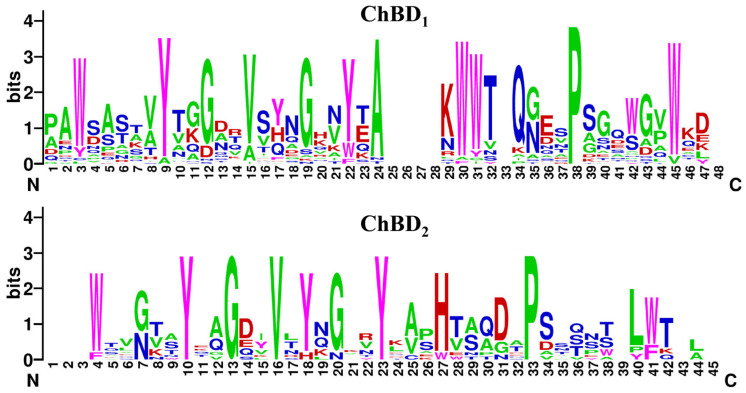
The conserved amino acid residues within the ChBD domains of *Ca*Chi18B are depicted using sequence logos for ChBD_1_, ChBD_2_, and the sequence of *Ca*Chi18B itself. These logos visually represent the amino acid composition and conservation at each position. Specifically, magenta represents aromatic amino acids, green indicates uncharged non-polar amino acids, red signifies charged polar amino acids, and blue represents uncharged polar amino acids.

### 2.2. Induction, Expression, and Purification of Recombinant Chitinase

**Figure 4:**
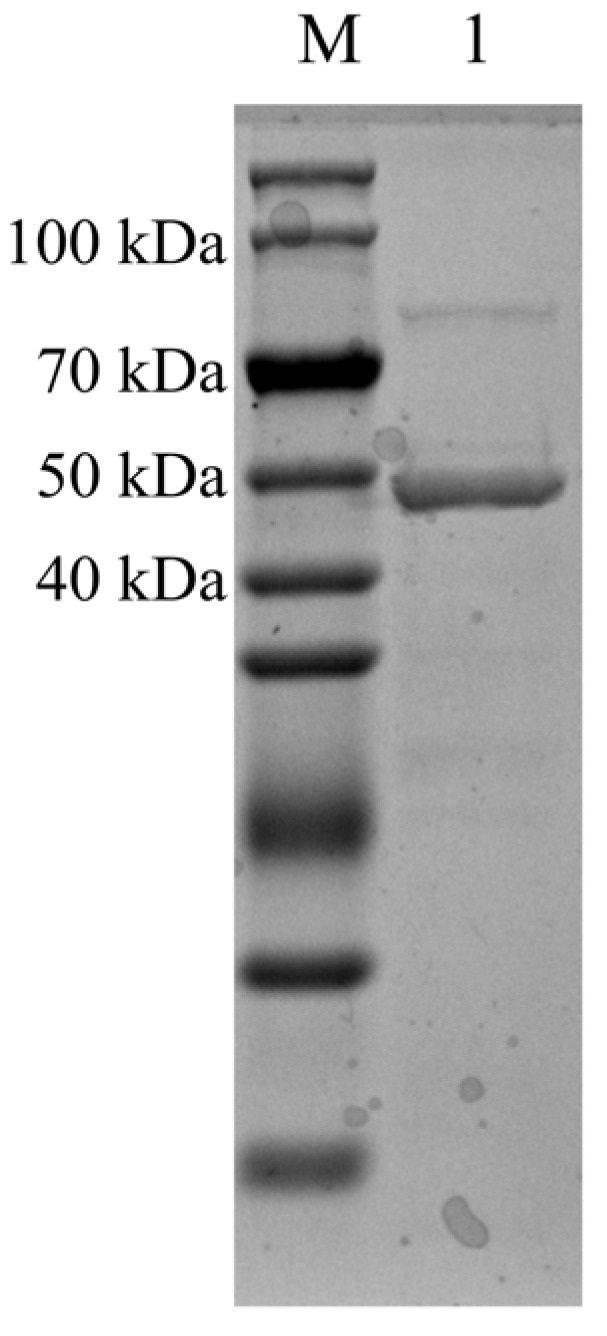
SDS-PAGE analysis of the recombinant *Ca*Chi18B_Δ ChBD_s_. M: Molecular mass markers (15–150 kDa). 1: Purified *Ca*Chi18B_ΔChBD_s_.

The target gene was ligated to the expression vector pET-22b (+), and the positive recombinant plasmid was transformed into the expression host *E. coli* BL21-CodonPlus (DE3)-RIPL. After purification using nickel affinity chromatography, SDS-PAGE analysis revealed a single protein band with a molecular weight of approximately 47 kDa (Figure 4), which was consistent with the theoretical molecular weight. The recombinant chitinase *Ca*Chi18B_ΔChBD_s_ was obtained, exhibiting an enzyme activity of 3.48 U/mg. This indicated that the protein was suitable for further studies on its enzymatic properties.

### 2.3. Analysis of the Enzymatic Properties of Recombinant Chitinase

#### 2.3.1. The Effects of Temperature and pH on the Activity of Recombinant Enzyme *Ca*Chi18B_ΔChBD_s_

Before assessing the effects of temperature and pH on the chitinase *Ca*Chi18B_ΔChBD_s_, we conducted elemental analysis on α-Chitin and colloidal chitin to determine their degree of acetylation based on the C/N ratio. It was found that the C/N ratio of colloidal chitin (C/N = 3.4156) was slightly higher than that of α-Chitin (C/N = 3.3493), indicating that acid hydrolysis does not significantly alter the degree of acetylation of chitin. Please refer to Figure S2 and Table S2 for further details.

**Figure 5:**
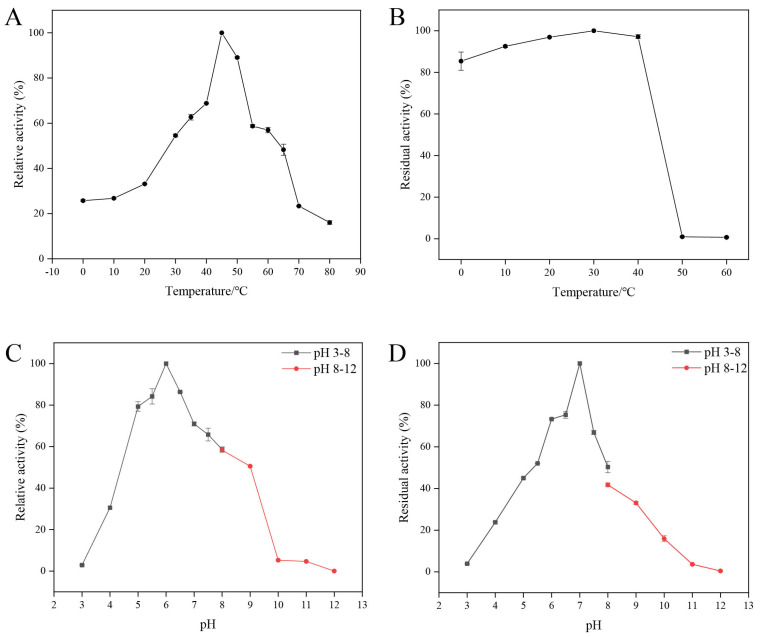
The effects of temperature and pH on the activity and stability of purified *Ca*Chi18B_ΔChBD_s_. (**A**) The optimal temperature of *Ca*Chi18B_ΔChBD_s_. (**B**) Temperature stability of *Ca*Chi18B_ΔChBD_s_. (**C**) The optimal pH of *Ca*Chi18B_ΔChBD_s_. (**D**) The pH stability of *Ca*Chi18B_ΔChBD_s_.

The optimal temperature for the recombinant chitinase *Ca*Chi18B_ΔChBD_s_ was 45 °C, as shown in Figure 5A. After incubation at temperatures ranging from 0 to 40 °C for 30 min, over 80% of the enzyme activity remained. However, at 50 °C, there was a sharp decline in residual enzyme activity.

The optimal pH was found to be 6.0, as illustrated in Figure 5C. The chitinase *Ca*Chi18B_ΔChBD_s_ retained more than 60% of its residual activity within the pH range of 6.0 to 7.5.

The optimal reaction temperature for *Ca*Chi18B_ΔChBD_s_ was 45 °C, and the optimal pH was 6.0, demonstrating slight variations in both the optimal temperature and pH compared to another truncated variant, *Ca*Chi18B_ΔChBD_1_ [38]; for instance, the chitinase Tc-ChiD from *Thermococcus chitonophagus* and its truncated variant Tc-ChiD (ΔCBD) exhibited a shift in temperature from 80 °C to 90 °C after truncation of the ChBD [42]. However, most ChBD_s_ do not influence the optimal temperature and pH of chitinases. For example, the optimal temperature and pH of the chitinase *Ca*Chi18A and its mutant *Ca*Chi18A_ChBD_s_, which originate from the same source, remain unchanged [40]. Similarly, the thermophilic chitinase ActChi retained an optimal temperature of 80 °C and a pH of 6.0 even after the addition of a ChBD [43]. In terms of stability, *Ca*Chi18B_ΔChBD_1_ exhibited greater temperature and pH stability than *Ca*Chi18B_ΔChBD_s_ [38].

#### 2.3.2. Determination of Enzyme Binding Ability

**Figure 6:**
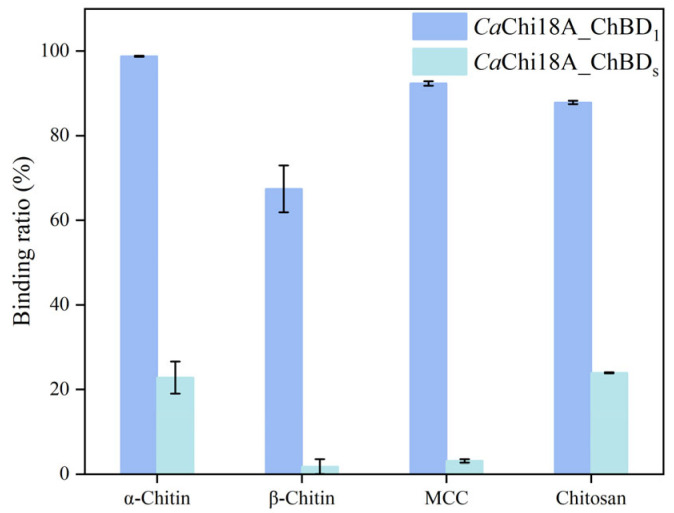
Binding abilities of *Ca*Chi18B_ΔChBD_1_ and *Ca*Chi18B_ΔChBD_s_ towards insoluble substrates.

Both *Ca*Chi18B_ΔChBD_1_ and its mutant *Ca*Chi18B_ΔChBD_s_ possess the ability to hydrolyze colloidal chitin into GlcNAc monomers [38]. Furthermore, this study also validated their binding potential with insoluble substrates, as clearly illustrated in Figure 6. The experimental results indicate that the presence of the ChBD significantly enhanced the binding affinity of chitinase for insoluble substrates. Specifically, when using α-Chitin, β-Chitin, microcrystalline cellulose (MCC), and chitosan as substrates, the binding rates of *Ca*Chi18B_ΔChBD_1_ were 98.88%, 71.32%, 98.14%, and 92.73%, respectively. In contrast, the binding rates of *Ca*Chi18B_ΔChBD_s_ for these substrates were only 25.52%, 2.3%, 3.3%, and 24.02%. Compared to *Ca*Chi18A_ΔChBD_1_, the binding rates were significantly reduced, demonstrating the critical role of ChBD in promoting the effective binding of chitinase to various substrates, in line with previous literature reports [30,31].

#### 2.3.3. Substrate Specificity and Kinetic Parameters of Recombinant Chitinase *Ca*Chi18B_ΔChBD_s_

To investigate the substrate specificity of *Ca*Chi18B_ΔChBD_s_, colloidal chitin, αChitin, β-Chitin, sodium carboxymethyl cellulose (CMC-Na), chitosan, and microcrystalline cellulose (MCC) were selected as substrates for the study, with the results summarized in Table 1. The data indicate that *Ca*Chi18B_ΔChBD_s_ exhibited the highest enzymatic activity on colloidal chitin, reaching 3.48 U/mg, followed by β-Chitin. However, when hydrolyzing insoluble substrates, *Ca*Chi18B_ΔChBD_s_ exhibited only 1.7% of its activity towards colloidal chitin on α-Chitin, whereas *Ca*Chi18B_ΔChBD_1_ achieved nearly double that at 3%. This aligned with the characteristics of many reported ChBD_s_, such as the fusion of the ChBD from *Bacillus subtilis* with the C-terminus of the chitinase Chit46, which enhanced the activity of the chimeric enzyme towards insoluble substrates by 219% [44]. Additionally, the chitinase Chi1 derived from *A. caviae* CB101 also exhibited reduced activity against MCC following truncation [45].

Using colloidal chitin as the substrate, the kinetic parameters of *Ca*Chi18B_ΔChBD_s_ were determined. Fitting calculations performed with GraphPad software yielded a Michaelis constant (*K_m_*) of 1.159 mg mL^−1^ and a maximum reaction rate (*V_max_*) of 10.37 μM min^−1^ mg^−1^. The catalytic constant (*k_cat_*) was found to be as high as 103.7 s^−1^. The *K_m_* value of *Ca*Chi18B_ΔChBD_s_ was 1.159 mg mL^−1^, whereas the *K_m_* value of *Ca*Chi18B_ΔChBD_1_ for colloidal chitin was 0.6626 mg mL^−1^. In comparison, *Ca*Chi18B_ΔChBD_1_ exhibited a lower *K_m_* value, indicating a higher affinity for the substrate [38]. It exhibited similarities to chitinases found in other sources, which demonstrated higher hydrolysis ability towards colloidal chitin but weaker hydrolysis ability towards powdered chitin [46,47,48]. Of interest, the enzyme also exhibited some activity when chitosan was used as a substrate, resembling the chitinases from *Orpinomyces* and *Anaeromyces* [49], but acting differently from the chitinases from *Streptomyces roseolus* [50] and *Paenicibacillus barengoltzii* [48].

#### 2.3.4. Tolerance of Recombinant Chitinase *Ca*Chi18B_ΔChBD_s_ to Metal Ions and Chemical Reagents

**Figure 7:**
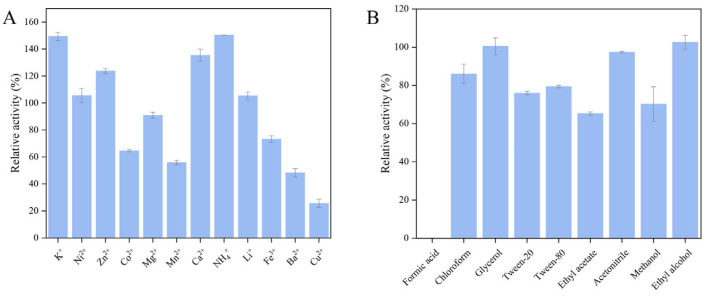
The effects of metal ions and organic reagents on the activity of *Ca*Chi18B_ΔChBD_s_. (**A**) Metal ion tolerance of *Ca*Chi18B_ΔChBD_s_. (**B**) Organic reagent tolerance of *Ca*Chi18B_ΔChBD_s_.

Metal ions such as K^+^, Ni^2+^, Zn^2+^, Ca^2+^, NH_4_^+^, and Li^+^ exhibited a promoting effect on the hydrolytic activity of *Ca*Chi18B_ΔChBD_s_, with K^+^ and NH_4_^+^ showing the most significant enhancement, increasing activity by up to 150% (Figure 7A). In contrast, metal ions such as Co^2+^, Mn^2+^, Fe^3+^, Ba^2+^, and Cu^2+^ had an inhibitory effect on *Ca*Chi18B_ΔChBD_s_, with Cu^2+^ demonstrating the most pronounced inhibition, resulting in only 23% of the enzyme’s activity remaining. Generally, Cu^2+^ is often utilized as an inhibitor for most chitinases derived from bacteria [51,52,53,54]. Recent research has revealed that Cu^2+^ may coordinate with the thiol side chain of cysteine residues within the active site of chitinases, thereby inducing conformational changes in the enzyme molecule and ultimately leading to the reduction or loss of enzymatic activity [51,55]. Research has found that a certain concentration of Co^2+^ can inhibit the activity of chitinases. Similarly, Co^2+^ also exhibited a strong inhibitory effect on chitinases from *Micrococcus* sp. AG84 [56] and *S. roseolus* [50].

Regarding organic reagents, while most had minimal impact on the enzyme activity of *Ca*Chi18B_ΔChBD_s_, formic acid was a notable exception, severely inhibiting the enzyme’s activity. This observation indicated that while the recombinant enzyme *Ca*Chi18B_ΔChBD_s_ behaves similarly to *Ca*Chi18B_ΔChBD_1_ [38], the activity of chitinase *Ca*Chi18B_ΔChBD_s_ is largely unaffected by most chemical reagents, with the exception of formic acid. Chloroform, Tween-20, Tween-80, glycerol, and ethyl alcohol have been shown to inhibit chitinase activity in most cases [51,57,58]. Interestingly, ethyl alcohol and glycerol do not affect the activity of chitinase *Ca*Chi18B_ΔChBD_s_, which also distinguishes it from other chitinases [57,58]. Lee et al. [53] initially reported an organic solvent-tolerant chitinase, named *Mt*Ch509, from the bacterium *Microbulbifer thermotolerans*. Notably, the presence of organic solvents, including benzene, dimethyl sulfoxide, hexane, isoamyl alcohol, isopropanol, and toluene (10–20%, *v*/*v*), significantly enhanced the reactivity of *Mt*Ch509.

#### 2.3.5. Analysis of Hydrolysis Products

**Figure 8:**
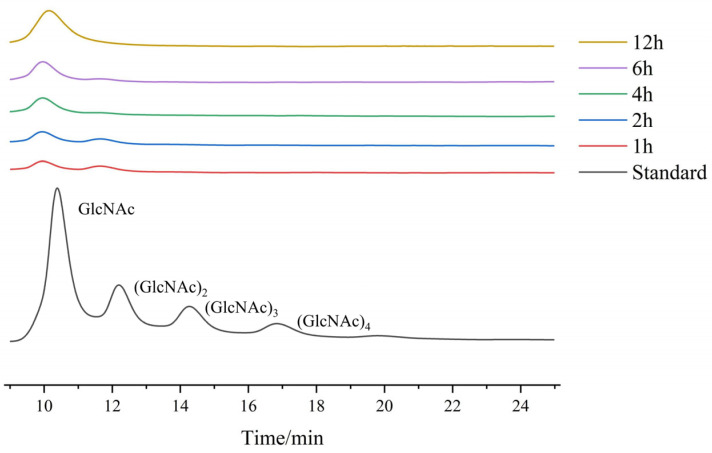
The product analysis of *Ca*Chi18B_ΔChBD_s_. *Ca*Chi18B was incubated with colloidal chitin at 30 °C for varying time periods. The reaction products were analyzed by HPLC. The peaks at the top represent a mixture of chitooligosaccharide standards: GlcNAc, (GlcNAc)_2_, (GlcNAc)_3_, and (GlcNAc)_4_.

High-performance liquid chromatography (HPLC) was employed to analyze the products generated by the recombinant enzyme *Ca*Chi18B_ΔChBD_s_ during the hydrolysis of colloidal chitin, as illustrated in Figure 8. The analysis of the hydrolysis products revealed that the primary products during the initial stages of the reaction were GlcNAc and (GlcNAc)_2_. However, as the reaction progressed, the products gradually shifted to GlcNAc alone, suggesting that *Ca*Chi18B_ΔChBD_s_ possesses both chitobiosidase activity and β-N-acetylglucosaminidase activity. In our research, we have discovered that the primary product of chitinase *Ca*Chi18B_ΔChBD_1_ during the initial stages of colloidal chitin hydrolysis was (GlcNAc)_2_, whereas the truncated variant *Ca*Chi18B_ΔChBD_s_ yielded GlcNAc as its initial product, potentially attributed to the truncated ChBD. A similar phenomenon was also observed in the chitinase *Bm*Chi, derived from *Bombyx mori*, and its truncated mutant *Bm*Chi∆C [59]. When hydrolyzing (GlcNAc)_5_, the product of chitinase *Bm*Chi was GlcNAc and (GlcNAc)_2_, whereas the product of *Bm*Chi∆C was (GlcNAc)_2_ and (GlcNAc)_3_. Additionally, when hydrolyzing (GlcNAc)_3_, the product of chitinase *Bm*Chi was GlcNAc and (GlcNAc)_2_, whereas the product of *Bm*Chi∆C, when hydrolyzing (GlcNAc)_5_, remained as (GlcNAc)_2_ and (GlcNAc)_3_ [60]. The removal of the C-terminal chitin-binding domain (ChBD) had a negative impact on the hydrolytic ability of *Bm*Chi towards trisaccharide substrates. This proves that ChBD has an influence on the distribution of the hydrolytic products of chitinase. Oligomers can pose a significant challenge in terms of separating the desired product and can create difficulties in their application during large-scale production [39,61]. This property may enable the potential application of *Ca*Chi18B and *Ca*Chi18A_ΔChBD_s_ in the field of green GlcNAc production.

## 3. Materials and Methods

### 3.1. Material and Reagents

#### 3.1.1. Strain and Plasmid

*C. aquatile* CSC-1, the expression vector pET-22b (+), *E. coli* DH5α, and *E. coli* BL21-CodonPlus (DE3)-RIPL were all preserved in the laboratory.

#### 3.1.2. Main Reagents and Instruments

The restriction enzymes *Nco* I and *Hin*d III were purchased from Takara Biomedical Technology (Tokyo, Japan) Co., Ltd. The Star Marker D2000, plasmid mini-prep kit, and DNA purification kit were obtained from Genstar (Suzhou, China). The 2× ClonExpress Mix and 2× Phanta Max Master Mix were sourced from Vazyme (Nanjing, China), while the Ni-NTA Beads 6FF for protein purification were acquired from Smart-Life Sciences (Changzhou, China) Co., Ltd.

The LB liquid culture medium consisted of 5 g/L yeast extract, 10 g/L peptone, and 10 g/L NaCl, sterilized at 121 °C for 20 min. The LB solid medium contained 5 g/L yeast extract, 10 g/L peptone, 10 g/L NaCl, and 15 g/L agar powder, also sterilized at 121 °C for 20 min. The PCR instrument used was the Biometra TOne, and the gel imaging system used was the Gel Doc™ XR+ from Bio-Rad (Hercules, CA, USA).

#### 3.1.3. Software and Websites

The physicochemical properties of the protein were analyzed using ExPASy [62] (https://web.expasy.org/compute_pi/, accessed on 6 July 2024). Signal peptide prediction was conducted using SignalP 6.0. [63] (https://services.healthtech.dtu.dk/services/SignalP-6.0, accessed on 1 July 2024). Domain analysis was performed using SMART (http://smart.embl.de/, accessed on 1 July 2024) [64]. MEGA 11 software was used to construct the phylogenetic tree based on the sequences [65]. ChiPlot was employed to beautify the phylogenetic tree (https://chiplot.online/, accessed on15 February 2025) [66]. WebLogo (http://weblogo.berkeley.edu/; WebLogo: A Sequence Logo Generator, accessed on 11 November 2024) was utilized to analyze the results of the multiple sequence alignments [67]. The three-dimensional structure was analyzed through the PyMOL program (The PyMOL Molecular Graphics System, accessed on 15 February 2025, Version 3.0 Schrödinger, LLC, New York, NY, USA).

### 3.2. Experimental Methods

#### 3.2.1. Preparation of Colloidal Chitin

Colloidal chitin was prepared according to previously established methods in the laboratory [40]. Ten grams of powdered chitin were mixed with 100 mL of concentrated hydrochloric acid and stirred until a homogeneous solution was achieved. The mixture was then allowed to stand at 4 °C for 24 h. Meanwhile, 2 L of industrial alcohol was pre-cooled. After 24 h, the chitin solution was combined with the industrial alcohol and stirred until homogeneous. The mixture was then allowed to stand at 4 °C for another 24 h, followed by centrifugation at 8000 rpm for 10 min to collect the precipitate, which was washed repeatedly with distilled water until neutralized.

#### 3.2.2. Gene Cloning and Construction of Expression Vectors for *Ca*Chi18B_ΔChBD_s_

Using the pET-22b (+) vector, primers for *CaChi18B_ΔChBD_s_*-F and *CaChi18B_ΔChBD_s_*-R were designed based on the gene sequence of *CaChi18B_ΔChBD*_s_. The primers included recognition sites for the restriction endonucleases *Nco* I (ccatgg) and *Hin*d III (aagctt), as shown in Table 2.

The PCR conditions were as follows: initial denaturation at 95 °C for 5 min, denaturation at 95 °C for 30 s, annealing at 65 °C for 30 s, and extension at 72 °C for 90 s, followed by 35 cycles of these steps. A final extension was performed at 72 °C for 10 min. The amplified DNA fragments were purified from the gel and then ligated into the pET-22b (+) plasmid, which had been double-digested with *Nco* I and *Hind* III and subsequently purified. The ligation mixture was transformed into competent *E. coli* DH5α cells and sent for sequencing. The recombinant plasmid was designated as pET-22b (+)-*CaChi18B_ΔChBD_s_*/DH5α.

#### 3.2.3. Protein Expression and Purification of *Ca*Chi18B_ΔChBD_s_

The correctly sequenced recombinant plasmid was introduced into the expression host *E. coli* BL21-CodonPlus (DE3)-RIPL.

After culturing the above strain for induction, the cells were collected by centrifugation at 8000 rpm for 10 min. The pellet was then resuspended in Tris-NaCl buffer and subjected to ultrasonic cell disruption to obtain the cell lysate. The lysate was centrifuged at 12,000 rpm for 30 min at 4 °C to collect the supernatant. Subsequently, affinity chromatography was performed using Ni-NTA Beads 6FF, and the elution fraction contained the recombinant enzyme.

#### 3.2.4. Enzymatic Property Analysis of *Ca*Chi18B_ΔChBD_s_

The colloidal chitin was frozen at −80 °C for 24 h, followed by lyophilization using a freeze dryer (YAMATO DC801). The α-Chitin was dried in an oven at 80 °C for 24 h. The dried materials were then analyzed using an elemental analyzer (Thermo Scientific Flash TY001, Waltham, MA, USA) to determine the C/N ratio, which was used to assess the degree of acetylation of the materials.

The activity of the chitinase was measured using the DNS method, with slight modifications based on the previous literature [40]. The reaction system consisted of 400 μL of 2% colloidal chitin (pH 7.0) and 100 μL of the enzyme solution at a concentration of 0.1 mg mL^−1^. The mixture was incubated at 50 °C for 10 min, followed by treatment in a boiling water bath for 10 min to terminate the reaction. The mixture was then cooled to room temperature in an ice-water bath. Subsequently, 500 μL of DNS solution was added to each tube, and the mixture was heated in a boiling water bath for 5 min to develop color, after which it was cooled to room temperature. The mixture was centrifuged at 12,000 rpm for 5 min, and 200 μL of the supernatant was transferred to a microplate for absorbance measurements at 540 nm. The blank control consisted of 100 μL of citrate-phosphate buffer and 400 μL of 2% colloidal chitin (pH 7.0) under the same reaction conditions. The yield of the reducing sugars was calculated based on the difference in absorbance between the experimental and control groups, and the chitinase activity was determined using a standard curve. Each experimental group was repeated three times. Chitinase activity (U) was defined as the amount of enzyme required to release 1 µmol of N-acetyl-D-glucosamine per minute under the specified reaction conditions. Specific activity (U/mg) is defined as the number of enzyme activity units present per milligram (mg) of chitinase.

##### Effect of Temperature on Recombinant Enzyme Activity

Diluted enzyme solutions were used to assess the impact of different temperatures on the activity of the recombinant enzyme, with 2% colloidal chitin as the substrate. The highest enzyme activity was set as 100%, and the relative enzyme activity at various temperatures was calculated to determine the optimal reaction temperature. The recombinant enzyme was incubated at different temperatures for 30 min, after which the residual enzyme activity was measured under optimal reaction conditions. The activity of the untreated pure enzyme was considered as 100%, allowing for the calculation of the residual enzyme activity of the recombinant chitinase after 30 min of incubation at each temperature. Each temperature group was performed in triplicate to ensure accuracy in the measurement of enzyme activity.

##### Effect of pH on Recombinant Enzyme Activity

The chitinase solution at a concentration of 0.1 mg/mL was mixed with 2% colloidal chitin in buffers ranging from pH 3.0 to 12.0 (100 mmol/L citrate-phosphate buffer: pH 3.0–8.0; glycine-NaOH buffer: pH 8.0-12.0), and the enzyme activity was measured at the optimal temperature to determine the optimal reaction pH. The maximum enzyme activity was set as 100%, and a curve was plotted based on the relative enzyme activity at different pH values. Each experimental group was performed in triplicate. The recombinant enzyme was incubated in buffers with varying pH (100 mmol/L citrate-phosphate buffer: pH 3.0–8.0; glycine-NaOH buffer: pH 8.0–12.0) at 4 °C for 180 min. Subsequently, 2% colloidal chitin (pH 6.0) was mixed with the incubated enzyme solution, and the enzyme activity was measured to evaluate the pH stability of the recombinant enzyme. Each pH condition was tested in triplicate to ensure the accuracy of enzyme activity calculations.

##### Determination of Enzyme Binding Capacity

The binding capacity of chitinase was assessed based on previous research by Chen et al. [40]. The substrates (α-Chitin, β-Chitin, MCC, and chitosan) were prepared at a concentration of 5% (W/V) in 100 mM citrate-phosphate buffer (pH 6.5). To achieve this, 500 μL of chitinase was added to 0.025 g of the substrate. The mixture was gently shaken at 4 °C for 1 h. After the reaction, the mixture was centrifuged to collect the supernatant, and the protein concentration was measured. The ratio of bound protein to the initial protein concentration was defined as the binding capacity of the enzyme to chitin.

##### Determination of Substrate Specificity, *K_m_*, and *V_max_*

The enzyme activity was measured using various substrates, including 2% colloidal chitin, α-Chitin, β-Chitin, sodium carboxymethyl cellulose (CMC-Na), chitosan, and microcrystalline cellulose, under optimal conditions.

Additionally, the enzyme activity of the recombinant enzyme was assessed using different concentrations (0.1–5%) of colloidal chitin as the substrate under optimal conditions. The data were plotted using GraphPad to calculate the *K_m_* and *V_max_* values.

##### Effects of Metal Ions and Organic Reagents on Recombinant Enzyme Activity

Diluted recombinant enzyme solutions were incubated with metal ions at a final concentration of 1 mmol/L (Co^2+^, K^+^, Zn^2+^, Ni^2+^, Mg^2+^, Mn^2+^, Ca^2+^, NH_4_^+^, Li^+^, Fe^3+^, Ba^2+^, Cu^2+^) and organic reagents at a final concentration of 1% (formic acid, chloroform, glycerol, Tween-20, Tween-80, ethyl acetate, acetonitrile, methanol, ethanol) under optimal conditions. The enzyme activity was measured, with the activity of the enzyme without any added metal ions or organic reagents set as 100%. The relative effects of various reagents on enzyme activity were then calculated.

##### Analysis of Hydrolysis Products

A mixture of 1 mL of the recombinant enzyme, at a concentration of 1 mg mL^−1^, and 3 mL of 2% colloidal chitin was incubated at 30 °C. Samples were taken at 0.5, 1, 2, 4, 6, 12, and 24 h, and the reaction was terminated by boiling for 10 min. After cooling in an ice bath, the mixture was centrifuged at 10,000 rpm for 2 min to collect the supernatant. The supernatant was then analyzed using high-performance liquid chromatography (HPLC). The chromatographic analysis was performed on a Luna^®^ 5 μm NH_2_ column (LC Column 250 × 4.6 mm), with a differential refractive index detector (RID). The mobile phase consisted of 70% acetonitrile, and the flow rate was set at 0.5 mL/min.

## 4. Conclusions

In summary, we obtained the gene product of *Ca*Chi18B_ΔChBD_s_ from *C. aquatile* CSC-1 and successfully cloned, functionally expressed, and biochemically characterized it. The optimal reaction temperature for the recombinant enzyme *Ca*Chi18B_ΔChBD_s_ was 45 °C, and the optimal pH was 6.0, with over 50% relative enzyme activity maintained within the pH range of 5.5 to 8.0. Metal ions such as Ca^2+^, K^+^, Zn^2+^, NH_4_^+^, and Li^+^ enhanced the enzyme’s activity, while Cu^2+^, Mn^2+^, Co^2+^, and Ba^2+^ exhibited significant inhibitory effects. When colloidal chitin was used as the substrate, the enzyme demonstrated the highest activity, with a Michaelis constant (*K_m_*) of 1.159 mg mL^−1^ and a maximum reaction rate (*V_max_*) of 10.37 μM min^−1^ mg^−1^. Analysis of the hydrolysis products revealed that *Ca*Chi18B_ΔChBD_s_ possesses dual functional activities as both a chitobiosidase and a β-N-acetylglucosaminidase, indicating its ability to directly produce GlcNAc. This finding provides an environmentally friendly method for the sustainable production of valuable chitin-derived products. Furthermore, the investigation of the ChBD confirmed its crucial role in substrate binding and product distribution, establishing a foundation for the modification and analysis of chitinases. Future research will focus on the impact of ChBD on product distribution.

## Figures and Tables

**Table 1:** Substrate specificity of *Ca*Chi18B_ΔChBD_s_.

Substrate	Relative Activity (%)
Colloidal Chitin	100
α-Chitin	1.71155 ± 0.09682
β-Chitin	10.37198 ± 1.24252
CMC-Na	0.3668 ± 0.03463
Chitosan	3.64104 ± 0.05201
MCC	ND

**Table 2:** Amplification primers of chitinase *Ca*Chi18B_ΔChBD_s_.

Primers	Sequence
*CaChi18B_ΔChBD_s_*-F	5′-cccagccggcgatggccatggCAGGTCGGCTCCTACTTCACG-3′
*CaChi18B_ΔChBD_s_-*R	5′-ctcgagtgcggccgcaagcttCTGCTTGACCTTGCTCATGGC-3′

The underlines indicate the restriction sites of Q. Cut *Nco* I and Q. Cut *Hin*d III: *Nco* I (ccatgg); *Hin*d III (aagctt).

## Data Availability

The data presented in this study are available on request from the corresponding author.

## Supplementary Materials

The following supporting information can be downloaded at: https://www.mdpi.com/article/10.3390/md23030093/s1, Table S1 Comparison of enzymatic properties of *Ca*Chi18B_ΔChBD_1_ and *Ca*Chi18B_ΔChBD_s_; Figure S1 The SDS-PAGE analysis of recombinant *Ca*Chi18B_Δ ChBD_s_; Figure S2 Elemental analysis spectra of α-Chitin and colloidal chitin.
