# Supplementary material for: The Impact of Chitinase Binding Domain Truncation on the Properties of CaChi18B from Chitinilyticum aquatile CSC-1
Source: Mar Drugs. 2025 Feb 20;23(3):93. doi: 10.3390/md23030093 (PMC11943626; doi:10.3390/md23030093)
Supplement: Supplementary file 1 [file marinedrugs-23-00093-s001.zip › marinedrugs-3473723-supplementary.pdf]

Supplementary material

Table S1 Comparison of enzymatic properties of *Ca*Chi18B_ΔChBD$_1$ and *Ca*Chi18B_ΔChBD$_s$

Comparison of enzymatic properties	Optimal temperature (℃)	Optimal pH	Temperature stability>80% (℃)	pH stability >60%	K_m (mg/mL)	V_{max} (μM $min^{-1}mg^{-1}$)	k_{cat} (s^{-1})
*Ca*Chi18B_ΔChBD$_1$	50	7.0	40	3-10	0.6626	1.104	11.04
*Ca*Chi18B_ΔChBD$_s$	45	6.0	40	6-7.5	1.159	10.37	103.7

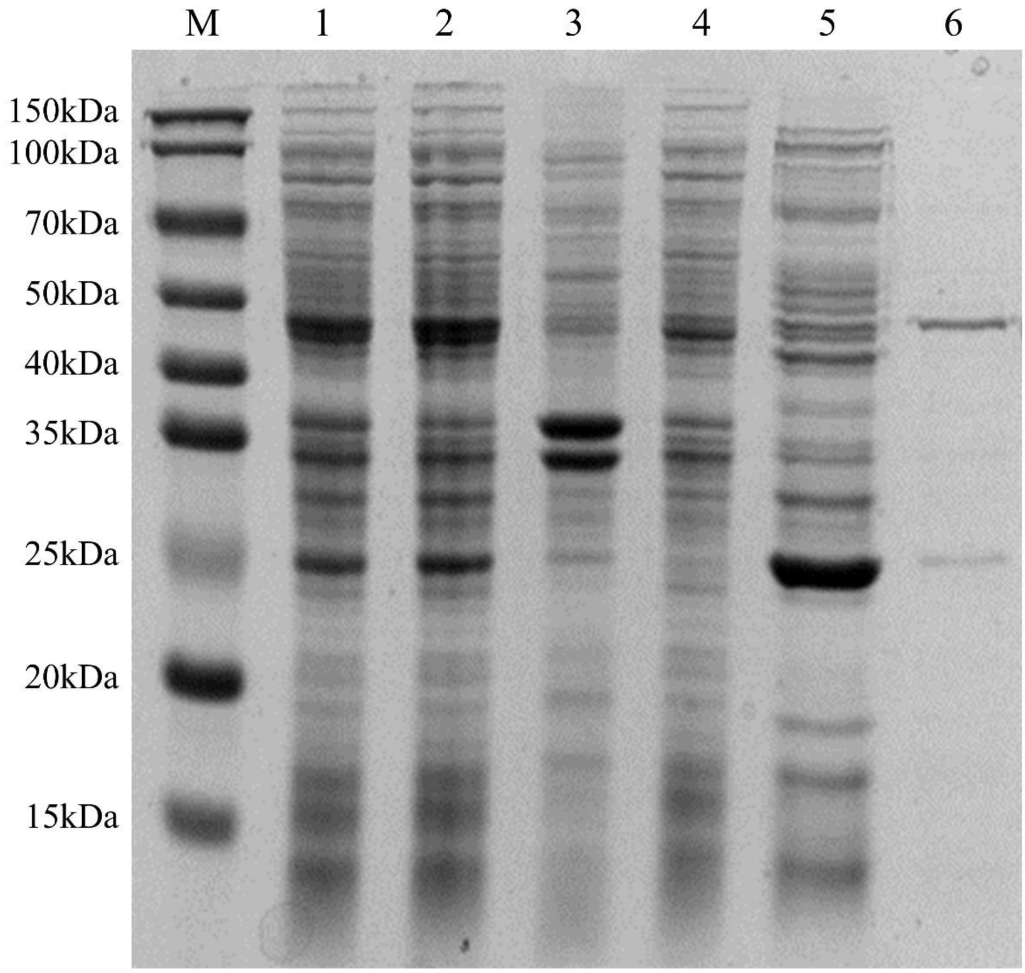

Fig S1 The SDS-PAGE analysis of recombinant *Ca*Chi18B_Δ ChBD$_s$ Lane 1, Cell lysates. Lane 2, the crude enzyme in the supernatant of lysates. Lane 3, the flow-through. Lane 4 and 5, the flow through of impurity protein eluted by 20 mM Tris-HCl buffer. Lane 6, purified protein eluate.

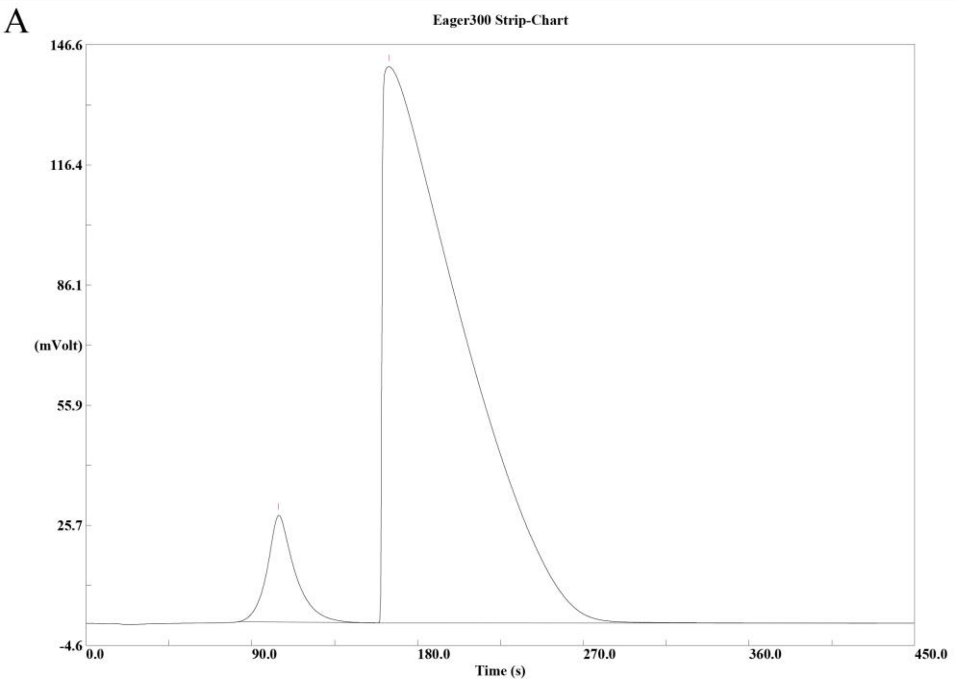

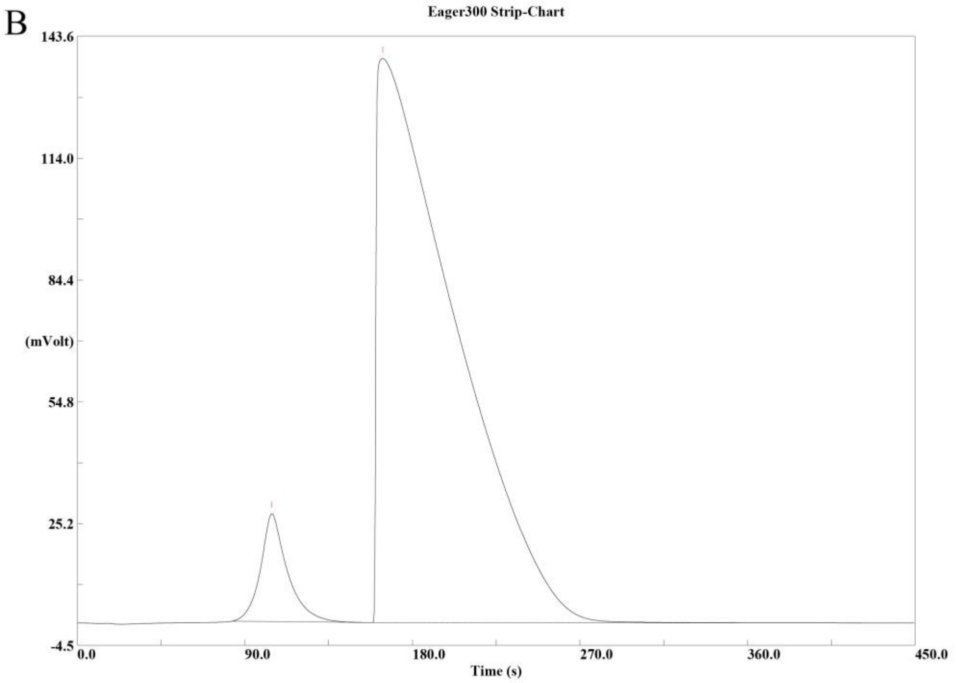

Fig S2 Elemental analysis spectra of α-Chitin and colloidal chitin (A) α-Chitin, (B) colloidal chitin.

Table S2 Elemental Analysis and C/N Ratio of α-Chitin and Colloidal Chitin

Sample Type	Sample Weight(mg)	C	N	C/N
α-Chitin	29.7	18.4276	5.3946	3.1416
colloidal chitin	30	19.5104	5.8252	3.3493
